# A new circulating accumulation emission model for assessing dust emission from open pit mine

**DOI:** 10.1038/s41598-021-03774-y

**Published:** 2021-12-20

**Authors:** Wanjun Tang, Fengming Li

**Affiliations:** 1 China Coal Research Institute, China Coal Technology and Engineering Group, Beijing, 100013 China; 2 Beijing Research Institute of Land Renovation and Ecological Restoration Technology, China Coal Technology and Engineering Group, Beijing, 100013 China

**Keywords:** Environmental impact, Engineering

## Abstract

To reduce the inaccuracy of using the monitoring data outside the pit to evaluate the unorganized emission dust source of open pit mine, the circulating accumulation emission model is established. Based on the model, the monitoring data in the pit can be converted into the dust emission from the pit. The main conclusions include: (1) the circulating accumulation emission model is suitable for the dust diffusion process in open pit mine. The ratio of diffusion $\mu$ and the ratio of surplus $\varepsilon$ were used to simulate the dust diffusion process in open pit mine, containing emission, retention and diffusion. (2) The initial value of the dust in the pit before the team operation has little influence on the final stable value. (3) When the external dust enters the pit, it will accumulate under the action of eddy current. The dust background value in the pit is different from that outside the pit. (4) The dust emission from the pit can be calculated from the monitoring data in the pit based on the circulating accumulation emission model. The model can deal with environmental changes such as the wind direction and speed, without arranging a lot of external monitoring equipment like the traditional external monitoring methods.

## Introduction

Surface mine is an unorganized dust source. At present, the monitoring method of unorganized dust emission intensity is to set up monitoring equipment separately in the upper and lower wind directions. The difference between the two types of monitoring data is the emission intensity of the unorganized dust source. However, due to the large area and varied shape of the open pit and a large number of dust sources in the pit, this value usually can not correctly reflect the dust pollution in the open pit in many cases. For example, when the wind direction or wind force changes, the upwind direction and downwind direction also change. When the shape of the mine pit and wind direction are in different combinations, the height of dust emitted outside the mine pit changes in a large range, etc. For this type of unorganized dust source of open pit, if we want to get the dust emission of the pit through the monitoring outside the pit, we need to arrange a large number of monitoring points in different positions and different elevations outside the pit, which is difficult to complete in practice.

According to the previous research results^1^, there is no significant difference in dust concentration in most spaces away from dust sources in the pit. The consistent law of dust distribution in the pit can be used to study the change rule of dust concentration in the mine pit, establish the dust diffusion model, and calculate the dust emission of the open pit mine.

As the monitoring data represents the accumulated value of dust emission, it may be unstable even though the emission keeps stable, because of dust cumulative process is affected by turbulence, which is easily affected by geometric dimension of open pit mine and weather conditions, such as wind, temperature, humidity et al.^2^. So the monitoring data cannot be directly used for assessing the dust emission intensity of mining activities.

In this study, the circulating accumulation emission model is established to convert the monitoring data in the pit into the dust emission of the pit. This method is less affected by wind direction, mine pit size and other factors. Hence the number of monitoring equipment needed is less. The dust emission assessment of open pit can be completed more efficiently.

## Dust diffusion in open pit mine

### Basic description of diffusion process

**Figure 1:**
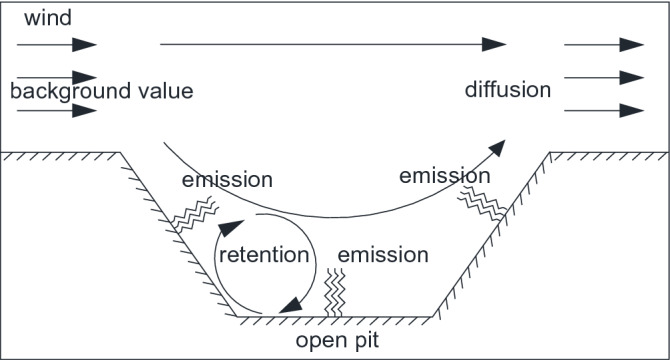
Dust diffusion process in open pit mine.

The monitoring data of particulate matter, abbreviated as PM, is composed of external transport dust, also called background value, and emissions by mining activity in open pit mine ^3^. As the effect of turbulence, which usually exists in open pit mine, some dust is retention in the pit and may float inside the pit for a long time. The other dust can escape from the pit and diffuses into the surrounding environment^1^, as shown in Fig. 1.

### Module of diffusion process

**Figure 2:**
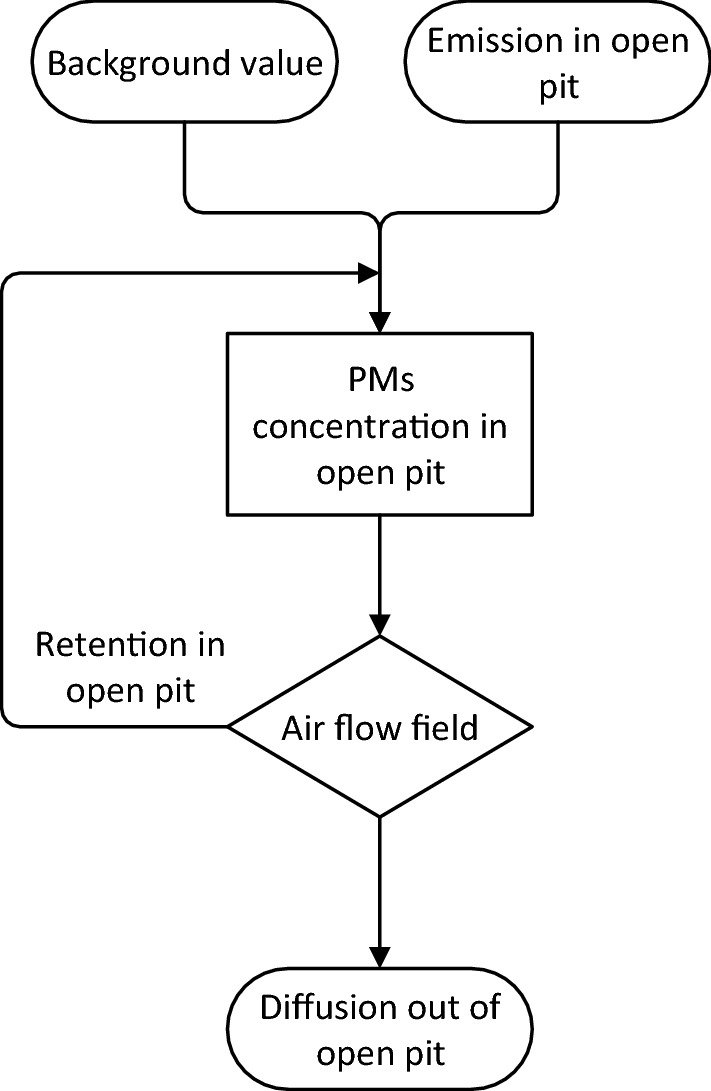
Module of diffusion process.

To model the diffusion process, a flow chart is used by decomposing the process, as shown in Fig. 2. This flow chart is the abstraction of the diffusion process shown in Fig. 1^4^. The meaning of them is similar. But based on the abstraction chart, the logical relationship among the parts of the diffusion process is clearer^5^. This relationship is consistent with the Markov birth and death process^6,7^.

This model analyzes the law of dust accumulation and diffusion in the mine pit under the influence of eddy current and its calculation model, so it is suitable for small particle size dust and not for large particle size dust. Therefore, it is not applicable to all PMs. It has good applicability to respirable dust, such as PM2.5, PM10, etc.

## The dust emission and diffusion model for open pit mine

### The basic form of model

To describe the diffusion process, time series analysis is suitable^8^. So this equation is used:

1 $$ Q(t) = Q(t - 1) + q(t) - \mu Q(t - 1) $$

where $t$ = the sequence of time, $Q(t)$ = the particles concentration in the open pit mine on the time $t$, $Q(t - 1)$ = the particles concentration in the open pit mine on the time $t - 1$, $q(t)$ = the emission on the time $t$, $\mu$ = ratio of diffusion to the external pit, $0 < \mu < 1$.

By arranging above equation, this equation is got:

2 $$ \begin{aligned} Q(t) & = (1 - \mu )Q(t - 1) + q(t) \\ & = \varepsilon Q(t - 1) + q(t) \\ \end{aligned} $$

where $\varepsilon$ = ratio of surplus in the pit, $0 < \varepsilon < 1$.

The variable $Q(t)$ is a time series of concentration of PMs. Derive the above equation by the variable $t$:

3 $$ \begin{aligned} Q(t) & = \varepsilon Q(t - 1) + q(t) \\ & = \varepsilon (\varepsilon Q(t - 2) + q(t - 1)) + q(t) \\ & = \varepsilon^{2} Q(t - 2) + \varepsilon q(t - 1) + q(t) \\ \end{aligned} $$

Further, recurse the equation by the variable $t$:

4 $$ \begin{aligned} Q(t) & = \varepsilon^{2} Q(t - 2) + \varepsilon q(t - 1) + q(t) \\ & = \varepsilon^{2} (\varepsilon Q(t - 3) + q(t - 2)) + \varepsilon q(t - 1) + q(t) \\ & = \varepsilon^{3} Q(t - 3) + \varepsilon^{2} q(t - 2) + \varepsilon q(t - 1) + q(t) \\ \end{aligned} $$

Finally, this equation is got by deriving the variable $t$ to 1:

5 $$ Q(t) = \varepsilon^{t} Q(0) + \varepsilon^{t - 1} q(1) + \varepsilon^{t - 2} q(2) + \cdots + \varepsilon^{t - j} q(j) + \cdots + \varepsilon q(t - 1) + q(t) $$

where $j$ = serial number of $q$, $0 < j < t$.

The above is the dust diffusion model for open pit mine. The variable $Q(0)$ represents the initial value of the concentration of PMs in open pit mine.

### A simplified form of model by dividing the operation time

It is unrealistic to obtain the real value of dust emission from mines with a general area of several square kilometers in real-time. So the model should be properly simplified so that it can not only calculate the dust emission but also ensure a certain accuracy. The basic idea of the simplified model in this paper is to segment the operation time according to the changes of influencing factors. In these divided time periods, since the influencing factors of the variable have not fluctuated greatly, it can naturally be considered that the variable has not fluctuated greatly. Since the main influencing factor of dust emission is the intensity and type of mining operation, the operation time can be segmented according to the actual operation intensity of the mine. In the divided operation time period, the electric shovel peels the coal and rock mass at the same position, the drilling rig drills at the same position, and the truck runs on the same road. Therefore, it can be assumed that the dust emission from mining operations in the pit has not changed during this time period. If the mining operation conditions change, the time period shall also be divided accordingly.

In the divided time period, the emission increment is assumed to be constant, which means that:

6 $$ q(1) = q(2) = \cdots = q(t - 1) = q(t) = q $$

The model is simplified:

7 $$ Q(t) = \varepsilon^{t} Q(0) + q(\varepsilon^{t - 1} + \varepsilon^{t - 2} + \cdots + \varepsilon + 1) $$

Arrange the equation:

8 $$ \begin{aligned} Q(t) & = \varepsilon^{t} Q(0) + q(\varepsilon^{t - 1} + \varepsilon^{t - 2} + \cdots + \varepsilon + 1) \\ & = \varepsilon^{t} Q(0) + q\frac{{1 - \varepsilon^{t} }}{1 - \varepsilon } \\ \end{aligned} $$

The above equation is the simplified form of model if the increase is constant.

As $0 < \varepsilon < 1$, the limit of $Q(t)$ is got:

9 $$ \mathop {\lim }\limits_{t \to \infty } Q(t) = \frac{q}{1 - \varepsilon } = \frac{q}{\mu } $$

The above equation shows that the model is convergent. The variable $\mathop {\lim }\limits_{t \to \infty } Q(t)$ represents the final steady state of the simplified model.

The limit of $Q(t)$ has nothing to do with $Q(0)$. In other words, whether the $Q(0)$ is bigger or smaller, with the same sequence of $q$, the final steady state of this simplified model, represented by $Q(t)$, is same.

### Application and parameters of the model in open pit mine

In the model, the variable $Q(j)$ is the result. But in monitoring activities, the variable $Q(j)$ is the monitoring data. The other variables should be calculated by $Q(j)$.

#### Background value

The background value is the concentration of PMs existing in the surrounding atmosphere. In the national standard, the background value should be monitored and subtracted for researching the pollution emission source^9^. However, due to the turbulence in the pit, not only the dust generated in the pit will accumulate, but also the dust in the surrounding environment outside the pit will accumulate after entering the pit. The dust emission is calculated by the method provided in this paper, and the detection data in the pit are used, which is the sum of the accumulated value of dust in the pit and the accumulated value of surrounding dust after entering the pit. Since the dust in the surrounding environment will accumulate and increase after entering the pit, its embodiment on the monitoring instrument in the pit is not equal to the concentration detected outside the pit. To eliminate the effect of background value, the monitoring data should be subtracted by the accumulated value, not the value monitored directly out of pit^10^.

Although the dust background concentration outside the pit is changing, it often remains stable for a period of time. Whether it fluctuates greatly can be judged by the consistency test. If the test is passed, the average value in this period shall be used instead. If the inspection fails, the monitoring data is segmented. Recheck whether the data in each segment meets the consistency test. Repeat the above operations until all segments of data meet the consistency test. In the divided time period, the dust background concentration $q_{back}$ is assumed to be constant.

By using the simplified model, when the variable $Q(0)$ is 0, the effect of background value in the pit is clear:

10 $$ Q_{back} (t) = q_{back} \frac{{1 - \varepsilon^{t} }}{1 - \varepsilon } $$

where $q_{back} (t)$ = the background value out of pit, $Q_{back} (t)$ = the effect of $q_{back} (t)$ in the pit.

The limit of $Q_{back} (t)$ is similar to limit of $Q(t)$.

11 $$ \mathop {\lim }\limits_{t \to \infty } Q_{back} (t) = \frac{{q_{back} }}{1 - \varepsilon } = \frac{{q_{back} }}{\mu } $$

If the fluctuation range of background value can be seen as constant, the limit of $Q_{back} (t)$ would be a better way to calculate the effect of background value in the pit.

If a mutation appears in the background value, the simplified model cannot work, the basic form of dust diffusion model should be used, and the effect of the mutation is calculated based on the Eqs. 3–5.

So, the concentration of PMs monitored in the pit should be handled by this equation:

12 $$ Q(t) = Q_{monitor} (t) - Q(t)_{back} $$

where $Q_{monitor} (t)$ = the concentration of PMs monitored in the pit.

The variable $Q(t)$, calculated by Eq. (12), can be dealt with by the diffusion model for understanding the rules and characteristics in an open pit mine after monitoring data obtained.

#### Ratio of surplus in the pit

The dust emission of open-pit mine is mainly caused by mining, blasting, transportation and dumping^11^. Although there is dust caused by natural wind, it accounts for a small proportion according to the research of previous scholars^12,13^. The premise of no dust emission proposed here is not that the wind speed is very small, but by analyzing the daily mining operation process of open-pit mine, select the time period when most equipment does not operate. During these non-operation periods, the manual operation is mostly stopped, and the dust generated by manual operation is ignored. During this period, the dust in the pit is gradually dissipated. The external wind speed is the same as the operation time period, that is, the diffusion conditions have not changed. Therefore, the dust monitoring data in the non-operation period can be used to calculate the diffusion coefficient, and the diffusion coefficient is also effective in the operation period with constant diffusion conditions. According to the law of mining operation, these non-operation periods occur many times every day. Such as daily maintenance period, daily evacuation period before blasting operation, shift handover period of different operation shifts, etc. The specific operation is to select one or more non-operation time periods according to the mining operation time arrangement, record the monitoring data and calculate the diffusion coefficient, which respectively represents the diffusion coefficient for a period of time. The escape rate calculated for these time periods acts on half of the adjacent time before and after them.

When $q(1) = q(2) = \cdots = (t - 1) = q(t) = 0$, which represents that there is no emission, the model is shown as below:

13 $$ Q(t) = \varepsilon^{t} Q(0) $$

Arrange the equation:

14 $$ \varepsilon = \sqrt[t]{{\frac{Q(t)}{{Q(0)}}}} $$

Then, a very important variable in this model is calculated out.

#### Value of emission

The variable $q(t)$ represents all emissions in the pit on the time $t$. Under the simplified model, the Eq. (9) can be used for calculating it. Arrange the Eq. (9):

15 $$ \begin{aligned} q & = (1 - \varepsilon )\mathop {\lim }\limits_{t \to \infty } Q(t) \\ & = (1 - \sqrt[t]{{\frac{Q(t)}{{Q(0)}}}})\mathop {\lim }\limits_{t \to \infty } Q(t) \\ \end{aligned} $$

So, all of variable in this model are calculated out.

The $q$ calculated by the Eq. (15) represents a mean value of emissions in the pit. Depend on it, the emission in a whole year can be calculated by accumulating the dust emission in all time periods:

16 $$ \begin{aligned} q_{year} & = \sum\limits_{{\text{t}}}^{T} {q*V} \\ & = \sum\limits_{{\text{t}}}^{T} {\left(1 - \sqrt[t]{{\frac{Q(t)}{{Q(0)}}}}\right)\mathop {\lim }\limits_{t \to \infty } Q(t)*V} \\ \end{aligned} $$

where $q_{year}$ = Annual emissions, $V$ = The air volume of open pit mine, $T$ = Annual working hours.

## Conclusions

Based on analyzing the accumulation and emission of dust concentration by dust monitoring data in an open pit, the circulating accumulation emission model is established. The advantage of the model constructed in this paper is to deduce the overall dust emission of the open-pit mine by analyzing the dust concentration change of the flow field in the pit.The model can deal with the change of environment, such as the change of wind speed and wind direction, without arranging a lot of external monitoring equipment like the traditional external monitoring methods. Because the traditional unorganized dust source monitoring method is used, a whole circle of dust monitoring equipment needs to be arranged around the mine to meet the monitoring conditions of different wind directions.

1. The model is suitable for the dust diffusion process in open pit mine, containing emission, retention, and diffusion.
2. The initial value of the dust in the pit before the team operation has little influence on the final stable value.
3. The dust background value of the surrounding environment monitored outside the pit can not be directly used. Because the dust of the surrounding environment may accumulate in the pit with turbulence. To eliminate the effect of background value, the monitoring data should be subtracted by the accumulated value, not the value outside the pit monitored directly.
4. Formula 16 can be used to calculate the dust emission in a certain monitoring period. If annual monitoring is maintained, the annual dust emission from an open pit can be obtained.
